# Neurocognitive Impairment in Severe Mental Illness. Comparative study with Spanish Speaking Patients

**DOI:** 10.3390/brainsci11030389

**Published:** 2021-03-19

**Authors:** Gabriel G. De la Torre, Sandra Doval, David López-Sanz, Manuel García-Sedeño, Miguel A. Ramallo, Macarena Bernal, Sara González-Torre

**Affiliations:** 1 Neuropsychology and Experimental Psychology Lab, University of Cadiz, 11510 Puerto Real, Spain; mantonio.garcia@uca.es (M.G.-S.); miguelangel.ramallo@gm.uca.es (M.A.R.); sara.gtm@uca.es (S.G.-T.); 2 Center for Biomedical Technology, Technical University of Madrid, 28040 Madrid, Spain; sandra.doval@ctb.upm.es (S.D.); david.lopez@ctb.upm.es (D.L.-S.); 3 Department of Experimental Psychology, Complutense University of Madrid (UCM), 28223 Madrid, Spain; 4 Department of Psychobiology and Methodology in Behavioral Sciences, Complutense University of Madrid (UCM), 28223 Madrid, Spain; 5 Tenpore Rehabilitation Center, 41011 Seville, Spain; bernalmaca@gmail.com

**Keywords:** neurocognitive, mental health, neuropsychology, Spanish, memory

## Abstract

Background. Serious mental illness (SMI) represents a category of psychiatric disorders characterized by specific difficulties of personal and social functioning, derived from suffering severe and persistent mental health problems. Aims. We wanted to look into differences in cognitive performance among different SMI patients. Methods. Repeatable Battery for the Assessment of Neuropsychological Status (RBANS) screening was applied in one sample of SMI patients (*n* = 149) and another of healthy comparison participants (*n* = 35). Within the SMI sample, three different subsamples were formed: one with 97 patients with schizophrenia, a second with 29 patients with mood disorders, and a third with 23 patients with personality disorder. We performed a comparative study within and between groups. Results. Analysis of covariance was performed. Significant differences were found for cognitive functioning including attention and memory. Conclusions. RBANS can be recommended for the detection of neurocognitive deficits in psychiatric disorders, especially in Schizophrenia.

## 1. Introduction

The concept of severe mental illness (SMI) is used to refer to a certain group of people with specific difficulties of personal and social functioning, derived from suffering severe and persistent mental health problems. We are, thus, referring to a group of people who require specific and preferential attention, given the multiplicity and severity of their problems [1]. Three dimensions are also considered when defining an SMI: clinical diagnosis, prolonged evolution over time (chronicity), and the level of social, family, and work disability [2].

According to the 10th revision of the International Statistical Classification of Diseases and Related Health Problems (ICD-10) [2], SMI diagnostic categories include psychotic symptomatology, severe alteration of interpersonal relationships, difficulties in the perception of reality, inappropriate affectivity and behaviour, and disorganized language. In ICD-10, SMI includes the following diagnostic categories: schizophrenic disorders (F20.x), schizotypal disorder (F21), persistent delusional disorders (F22), induced delusional disorders (F24), schizoaffective disorders (F25), other non-organic psychotic disorders (F28 and F29), bipolar disorder (F31.x), severe depressive episode with psychotic symptoms (F32.3), severe recurrent depressive disorders (F33), and obsessive-compulsive disorder (F42). According to the Diagnostic and Statistical Manual of Mental Disorders [3], serious mental illnesses include schizophrenia spectrum disorders, severe bipolar disorder, and severe major depression. However, when other mental illnesses cause significant functional impairment and substantially limit major life activities, they can also be considered to be a serious mental illness. In line with this description, in this study we studied neurocognitive profiles in three different groups of severe mental illness patients: schizophrenia patients, depression and bipolar disorder patients, and patients with severe cases of personality disorders representative of chronic severe mental illness.

Although all cases mentioned above present diverse symptoms, they usually share neurocognitive impairment, in addition to the presence of neurobiological vulnerability and social maladjustment [4,5,6,7]. It has been demonstrated that a high percentage of people suffering from SMI show poor performance in different aspects of cognitive processing, such as processing speed, maintenance of attention, working memory, verbal and learning skills, or social cognition [8,9,10,11].

Neuropsychological assessment in SMI has been relegated to the background because of the lack of specific instruments to measure it, especially for Spanish speaking patients due to several reasons including lack of validated, translated tests. This problem also affects to neuropsychological assessment of Spanish speaking population in the United States [12]. However, some neuropsychological instruments exist that—although they were developed to evaluate other conditions such as dementia—may represent a valid and helpful tool in the detection of cognitive impairment in SMI patients and they have been adapted to Spanish: The Screen for Cognitive Impairment in Psychiatry (SCIP) [13], the Epidemiological Study of Cognitive Impairment in Schizophrenia Battery (EPICOG-SCH) [14] and the Measurement and Treatment Research to Improve Cognition in Schizophrenia (MATRICS) developed by National Institute of Mental Health (NIMH) and adapted and standardized in Spain by Rodriguez-Jimenez, Bagney, Garcia-Navarro, Aparicio, Lopez-Anton, Moreno-Ortega and Green [15]. However, all of them have in common that they are brief screening tests while the Repeatable Battery for the Assessment of Neuropsychological Status (RBANS) represents a more comprehensive assessment tool, already tested in many different pathologies and adapted worldwide. Given the lack of instruments adapted for the Spanish-speaking population in this field, we considered interesting to test the possible usefulness of the version adapted to Spanish of RBANS.

RBANS was developed primarily for identification and assessment of neurocognitive impairment in dementia [4,16,17,18], but its efficacy has been proven in other types of conditions such as schizophrenia [19,20,21].

In this study, we used a Spanish version of RBANS Form A to compare the results among three different groups of psychiatric disorders and a group of healthy participants. The three experimental groups were comprised of patients with severe mental illness (SMI), including schizophrenia, depression and bipolar, and personality disorders. These psychiatric disorders may have a different impact in the neurocognitive status.

Schizophrenia usually presents cognitive deficits, some of which involve dorsolateral prefrontal cortex dysfunction [22]. Neurophysiological findings in schizophrenia also show evidence of pyramidal cell dendritic atrophy, likely reductions in cortical dopamine, and possible changes in dopamine D1 receptors [23]. The cognitive deficits present in the course of schizophrenia are related to verbal memory [24], and they have multiple neuropsychological deficits in tests of complex conceptual reasoning, psychomotor speed, new learning and incidental memory, and both motor- and sensory-perceptual abilities [25]. There are dysfunctions in motor and processing speed and emotion recognition [26]. Some such deficits appear to predate clinical symptoms and are exacerbated by typical illness onset during late adolescence or early adulthood [27,28].

Repeated self-damaging behaviour occurring in the context of borderline personality disorder (BPD) may reflect impairments in decision-making and planning cognition [5]. The RBANS has also been used with patients with personality disorder, obtaining a significantly worse performance in the cognitive measures in cluster B evaluated through this test [29].

Major depressive disorder (MDD) is a relatively common condition with high rate of recurrence and chronicity with clear effects on disability [30]. Manifestations of cognitive deficits can be different across patients with MDD. Previous research has shown that several regions of the brain may be affected in MDD including the hippocampus where its size has been demonstrated to be inversely correlated with illness duration [31]. Cognitive deficits in MDD may improve with treatment, but these deficits can still be detected in periods of symptom remission [32]. The RBANS has been tested with success in MDD patients to detect these cognitive deficits [33].

Bipolar disorder (BD) is a severe psychiatric illness that has been ranked as one of the 20 leading medical causes of disability [2]. Aside from executive function, multiple other facets of cognition have been widely studied in bipolar disorder using specific neuropsychological tests such as the trail making test (TMT), verbal fluency [34], and RBANS; it was discovered that the RBANS shows deficits in the total score, immediate and delayed memory, and visuospatial ability [35,36].

There is strong evidence that depression is associated with neuropsychological deficits across multiple domains [33]. RBANS has also successfully been used to look into medical comorbidity impact in cognitive processing in depression patients [37] and in the detection of mild cognitive impairment (MCI) in patients with depression [38].

The objectives of this study were first to evaluate the presence of neurocognitive deficits in a sample of patients with SMI compared with another equivalent sample of healthy participants. Secondly, we discuss the different specific neurocognitive profiles of each disorder evaluated. Finally, we check the utility of the RBANS to detect the presence of this symptomatology. Some research using RBANS has shown that social and employment adjustment of these patients is very important for their prognosis and detecting cognitive deficits can be of help [39] Results of this study may help to support the use of RBANS as a helpful tool to properly detect cognitive deficits in SMI patients and to choose better treatment options for optimum personal and social adjustment.

## 2. Materials and Methods

### 2.1. Participants

Potential participants for this study were excluded if, in addition to their psychiatric diagnosis, they had any type of central nervous system (CNS) disease affecting cognition and/or functional abilities, a history of CNS infections, a history of or current alcohol or drug abuse, or if they presented with other characteristics that rendered their participation inappropriate for this research. Selection was performed based in chart diagnosis certified by clinical psychologists or psychiatrists at the patients’ medical centres and associations not affiliated with this study. The control group was formed by persons without any mental disorder or physical illness and who had no history of serious physical or psychological disorders. Education level (number of years of school attendance) and gender information were collected to verify a homogeneously distributed representative sample according to the Spanish census. Mean educational level (measured from 1—illiterate to 7—postgraduate studies) was similar in the different groups (F = 0.7, *p* = 0.557) (Control: mean = 3.66, sd = 0.53; Schizophrenia: mean = 3.42, sd = 0.95; Mood: mean = 3.59, sd = 0.98; Personality: mean = 3.52, sd = 1.08;). Participants in both samples (SMI and healthy comparison group) were aged between 20 and 59 years and had at least basic literacy skills, good hearing, and visual and verbal capacity to perform the tests.

The recruiting effort was disseminated through the communication channels existing between different registered organizations and associations of mental health patients in the southern region of Spain that willingly participated without remuneration. All participants were clearly informed about the tests and were asked to express verbal or written consent. This research was completed in accordance with the Helsinki Declaration.

The participants included 149 patients with an SMI (101 males, 48 females) and a control group which consisted of 35 healthy participants without any psychological pathology. The SMI group was divided into three subgroups according to diagnosis categories of Diagnostic and Statistical Manual of Mental Disorders 4th edition text revision (DSM IV-TR) [40]. The first group was called “Schizophrenia” (*n* = 97) and this group consisted of patients with schizophrenia; the second group, “Mood” (*n*= 29), included patients within the DSM IV-TR category of Mood Disorders. For our sample: depression (*n* = 12) and bipolar disorder (*n* = 11). The third one, “Personality Disorders” (*n* = 23), included borderline personality disorder patients.

### 2.2. Procedure

Prior to the assessment using the RBANS (Form A), demographic data (including age, gender, and years of formal education) were collected for both groups. The RBANS is a brief neurocognitive battery with four alternative forms that assesses immediate and delayed memory, attention, language, and visuospatial/constructional skills. The RBANS is a brief neuropsychological battery that has been used for the detection of cognitive impairment in degenerative and non-degenerative neurological diseases [4,41,42,43,44]. The RBANS requires approximately 20–30 min to administer. The tests in RBANS are based on traditional neuropsychological tasks. The RBANS generates six different index scores—a total scale index and five specific scoring indices that assess immediate memory, visuospatial/constructional skills, language, attention, and delayed memory and its form A has been validated and normative data obtained for Spanish speaking population in Spain [4,16]. Although the RBANS has four alternative forms, we only used Form A in our study because the existing normative data is available for this form only [4,16,45]. This is currently the most frequently used form in Spanish. The validity of cross-national neuropsychological assessment with the RBANS has been supported in studies of psychiatric disorders [35,46] and the overall reliability coefficient (Cronbach’s alpha) for the Spanish form A is 0.92 [4]. The RBANS form A used in this study was the validated and translated version for Spain [4,16]. Different versions exist for other Spanish speaking populations such as Mexican American [43].

To analyse the data, we used MATLAB for graphs and SPSS version 21.0 software for statistics (IBM, Armonk, NY, USA). First, we carried out a descriptive statistical data analysis. Groups did not significantly differ regarding age (F_age_ = 2.46, *p*_age_ = 0.290). However, they did differ with respect to sex (*p*_sex_ < 0.001). Effect sizes were calculated using eta-squared index. With the aim of evaluating between-group differences in the RBANS subtests, an ANCOVA analysis was conducted, including sex as a covariate, using 10.000 random diagnostic reassignment of our participants to obtain a non-parametric *p*-value due to the non-normality of our data (α = 0.05). In order to correct multiple comparisons, the false discovery rate (FDR) method was applied (*q* = 0.05) for each of the 19 RBANS subtests performed [47]). Furthermore, post hoc comparisons to test pair group differences were corrected using Tukey’s HSD method.

## 3. Results

Demographic data regarding group composition is shown in Table 1. We performed an ANCOVA analysis to look into between group differences (Table 1).

### 3.1. Between-Group Comparison

We observed a significant main effect of diagnostic on cognitive performance for the overall performance of the test as well as for all the subtests of the RBANS excluding picture naming, digit span, list recall, and visuospatial construction. A complete list of the F and *p* values for the subtests can be found in Table 1. Effect sizes (eta squared η^2^) varied from medium to high in the subtests showing a significant effect of diagnostic. In those comparisons in which no significant differences were found, the effect size was low. The complete set of effect sizes is reported in Table 1.

### 3.2. Control vs. Schizophrenia Disorder

The control and schizophrenia groups differed significantly in RBANS total performance and in all the subtests for which a main effect of diagnostic was observed.

### 3.3. Control vs. Mood Disorder

**Figure 1:**
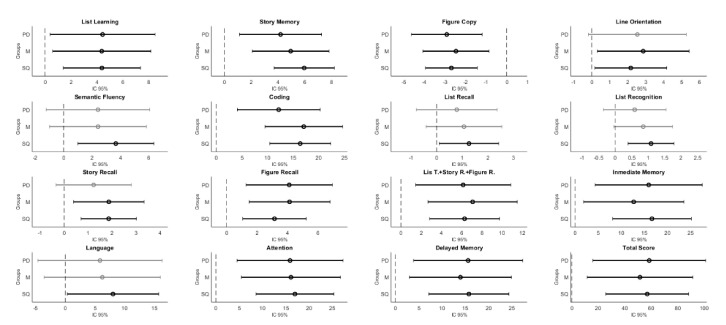
*PD:* Borderline Personality Disorder; *M*: Mood Disorder; *SQ*: Schizophrenia. Significant confidence intervals after multiple comparison correction are shown in these figures. Black bold lines intervals represent those comparison between control group and pathological group that reminds significant, whereas grey ones, represent those in which there are not significant differences. The middle point shows the mean difference of intervals.

The control and mood disorder groups did not significantly differ in semantic fluency and language subtests, whereas there were statistically significant differences in total performance and in the rest of the subtests and indexes tested for post hoc comparisons, as shown in Table 1 and Figure 1.

### 3.4. Control vs. Personality Disorder

Post hoc comparisons revealed that the control and personality disorder group did not significantly differ in line orientation, semantic fluency, list recognition, story recall and language subtests. However, there were statistically significant differences in total performance and the rest of subtests (see Table 1).

### 3.5. Comparison among Clinical Groups

Clinical groups (schizophrenia, mood disorder, and personality disorder) did not differ in any of the subtests in the post hoc comparisons.

### 3.6. Global Performance Comparison

**Figure 2:**
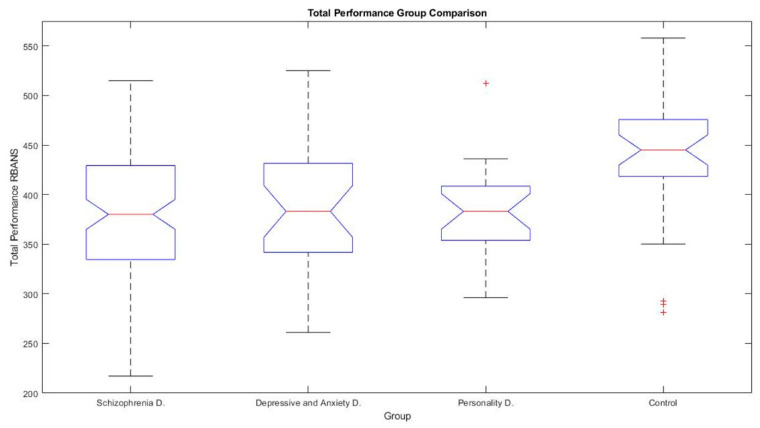
Boxplot showing total performance of each group in Repeatable Battery for the Assessment of Neuropsychological Status (RBANS) total score.

There is a significant effect of diagnostic on the global performance of the test (*F* = 8.31, *p* = 8.78 × 10^−5^). Post hoc comparisons showed that healthy comparison group performed significantly higher than the pathological groups. Healthy comparison group obtained the highest global performance (440 ± 66.65), followed by Mood Disorder patients (388.52 ± 64.32), Schizophrenia Disorder group (383.04 ± 58), and Personality disorder group (381.52 ± 46.14). However, no statistically significant differences were found between pathological groups in the total performance (Figure 2).

## 4. Discussion and Conclusions

The results show that Schizophrenia was the group with the most significant differences from the healthy comparison group. These results are in line with previous research where schizophrenia has been shown to have neurocognitive deficits in different domains [19,21,48,49]. The RBANS allowed us to concretize and locate the specific areas where this impairment can be found. As for the other two disease groups (Mood and Personality) we found less clear and less significant differences with control participants, but some commonalities were acknowledged. In general, language seems to be preserved in the Mood and Personality groups, unlike in the Schizophrenia group. Language-related problems detected by RBANS are in line with previous research on the topic [50,51]. Attention, visuospatial, some executive, and especially memory problems were common for all disease groups in the study, according to the results. Recent systematic reviews and research on executive and other cognitive deficits in borderline personality disorder [52], bipolar disorder [53], depression [54,55,56], and Schizophrenia [57,58] confirm these RBANS findings. RBANS is an especially sensitive tool for the detection of memory-related problems because these symptoms are common in dementia, and this was the original target population of the battery. In our study, RBANS was able to clearly detect memory problems of all types (short, working, and delayed) in the Schizophrenia group against healthy controls or healthy comparison group. However, the results for the other two groups, Mood and Personality, should be taken with caution because of the small size of the groups, constituting this a limitation of the study. Further research is needed in this direction with larger samples for these specific categories.

The trend towards differences in sex distribution between groups is a potential limitation of this study. Future studies should further address this issue. Remarkably, this study proves the helpfulness of the RBANS in the detection of neurocognitive or neuropsychological problems in psychiatric patients, particularly in SMI cases in a transversal comparative study among three different clinical samples, confirming previous research carried out with single and non-severe forms of the same diseases [59,60,61,62]. Despite some efforts having been made to adapt and validate this battery to Spanish speaking populations in Spain and United States [4,16,43], further research is needed to adapt the RBANS to the different Spanish speaking countries due to possible language variations.

In light of these results, we consider that the Spanish version of RBANS constitutes a helpful tool for the detection of neurocognitive deficits in SMI patients and also for establishing neuropsychological specific profiles that can be used in benefit of a more holistic rehabilitation and treatment approach of Spanish speaking patients.

## Figures and Tables

**Table 1:** Healthy Controls (*n* = 35), Schizophrenia (*n* = 97), Mood Disorder (*n* = 29), and Borderline (BL) Personality Disorder (*n* = 23). Mean ± standard deviation information is shown in the left part of the table. ANCOVA *p*-values after FDR correction (*q* = 0.05) are shown in the middle and post hoc *p*-values on the right part for each between-group comparison. Significant *p*-values were marked with an asterisk.

	MEAN ± STD				ANCOVA		Post hoc *p*-Values			Effect Size
	Healthy Controls	Schizophrenia Disorder	Mood Disorders	BL Personality Disorder	ANCOVAF	*p*-Value	Controls vs. Schizophrenia	Controls vs. Mood	Controls vs. Personality	Eta-Squared (η^2^)
*n*	35	97	29	23						
Age	38.63 ± 17.98	40 ± 7.45	42.48 ± 8.83	37.22 ± 7.44						
Gender (M-F)	11/24	69/28	16/13	16/7						
Level of education	3.66 ± 0.53	3.42 ± 0.95	3.59 ± 0.98	3.52 ± 1.08						
Total performance	440 ± 66.65	383.04 ± 58	388.52 ± 64.32	381.52 ± 46.14	8.31	<0.001 *	<0.001 *	0.002 *	0.002*	0.108
List Learning	28.8 ± 6.89	24.40 ± 5.52	24.41 ± 5.29	24.35 ± 4.44	4.84	0.004 *	0.002 *	0.016 *	0.024 *	0.063
Story Memory	17.37 ± 3.93	11.4 ± 4.5	12.41 ± 4.52	13.17 ± 3.58	16.6	<0.001 *	<0.001 *	<0.001 *	<0.001 *	0.196
Figure Copy	16.26 ± 3.8	18.95 ± 2	18.72 ± 2.07	19.17 ± 1.40	11.82	<0.001 *	<0.001 *	<0.001 *	<0.001 *	0.156
Line Orientation	17.46 ± 3.49	15.28 ± 3.81	14.59 ± 4.26	14.91 ± 3.67	5.96	0.001 *	<0.001 *	0.002 *	0.01 *	0.060
Picture Naming	9.63 ± 1	9.75 ± 0.9	9.62 ± 0.78	9.87 ± 0.34	0.27	0.85	0.99	0.99	0.92	0.007
Semantic Fluency	18.94 ± 6.58	15.26 ± 4.63	16.52 ± 5.41	16.53 ± 3.93	3.74	0.02 *	0.005*	0.28	0.47	0.064
Digit Span	8.2 ± 1.65	7.86 ± 2.07	7.52 ± 1.79	7.74 ± 2.99	1.16	0.37	0.41	0.33	0.9	0.009
Coding	47.83 ± 14.47	31.45 ± 10.12	30.76 ± 11.81	35.65 ± 9.76	15.94	<0.001 *	<0.001 *	<0.001 *	0.003 *	0.168
List Recall	6.49 ± 1.01	5.22 ± 2.5	5.41 ± 2.20	5.70 ± 2.22	2.18	0.11	0.06	0.29	0.68	0.04
List Recognition	19.86 ± 0.35	18.76 ± 1.6	19.00 ± 1.36	19.26 ± 0.92	5.04	0.003 *	<0.001 *	0.066	0.38	0.075
Story Recall	7.63 ± 0.64	5.76 ± 2.38	5.76 ± 2.61	6.39 ± 2.31	6.56	<0.001 *	<0.001 *	0.003 *	0.09	0.094
Figure Recall	15.91 ± 3.37	12.75 ± 3.87	11.76 ± 4.35	11.78 ± 4.66	8.23	<0.001 *	<0.001 *	<0.001 *	<0.001 *	0.119
List T. + Story R. + Figure R.	30.03 ± 4.17	23.73 ± 6.8	22.93 ± 7.05	23.87 ± 7.91	8.88	<0.001 *	<0.001 *	<0.001 *	0.003 *	0.125
Inmediate Memory	90.40 ± 18.49	73.78 ± 15.84	77.72 ± 17.31	74.48 ± 11.82	8.66	<0.001 *	<0.001 *	0.01 *	0.002 *	0.112
Visuospatial	88.77 ± 18.49	89.18 ± 17.23	86.24 ± 17.49	83.57 ± 17.94	0.58	0.66	0.99	0.9	0.71	0.011
Language	91.54 ± 18.91	83.52 ± 13.02	85.31 ± 15.65	85.70 ± 11.56	3.54	0.02 *	0.06	0.2	0.31	0.062
Attention	76.83 ± 17.54	59.87 ± 15.08	60.72 ± 14.46	60.91 ± 18.03	9.53	<0.001 *	<0.001 *	<0.001 *	0.004 *	0.109
Delayed Memory	92.46 ± 6.14	76.7 ± 18.03	78.52 ± 18.89	76.87 ± 16.01	7.54	<0.001 *	<0.001 *	0.004 *	0.003 *	0.111

## Data Availability

Restrictions apply to the availability of these data. Data was obtained from FAISEM and are available from www.faisem.es (accessed on 20 January 2021) with the permission of FAISEM.
